# Genome-Wide Association Analysis of Yield-Related Traits and Candidate Genes in Vegetable Soybean

**DOI:** 10.3390/plants13111442

**Published:** 2024-05-23

**Authors:** Hongtao Gao, Guanji Wu, Feifei Wu, Xunjun Zhou, Yonggang Zhou, Keheng Xu, Yaxin Li, Wenping Zhang, Kuan Zhao, Yan Jing, Chen Feng, Nan Wang, Haiyan Li

**Affiliations:** 1 School of Breeding and Multiplication (Sanya Institute of Breeding and Multiplication), Hainan University, Haikou 572025, China; 184240@hainanu.edu.cn (H.G.); 18108531479@163.com (G.W.); wff665@hainanu.edu.cn (F.W.); zhou1213155@163.com (X.Z.); ygzhou@hainanu.edu.cn (Y.Z.); xh312319@163.com (K.X.); yaxinli@hainanu.edu.cn (Y.L.);; 2 Changchun Academy of Agricultural Science, Changchun 130118, China

**Keywords:** vegetable soybean, plant height, agronomic trait, fresh pod height, GWAS

## Abstract

Owing to the rising demand for vegetable soybean products, there is an increasing need for high-yield soybean varieties. However, the complex correlation patterns among quantitative traits with genetic architecture pose a challenge for improving vegetable soybean through breeding. Herein, a genome-wide association study (GWAS) was applied to 6 yield-related traits in 188 vegetable soybean accessions. Using a BLINK model, a total of 116 single nucleotide polymorphisms (SNPs) were identified for plant height, pod length, pod number, pod thickness, pod width, and fresh pod weight. Furthermore, a total of 220 genes were found in the 200 kb upstream and downstream regions of significant SNPs, including 11 genes encoding functional proteins. Among them, four candidate genes, *Glyma.13G109100*, *Glyma.03G183200*, *Glyma.09G102200*, and *Glyma.09G102300* were analyzed for significant haplotype variations and to be in LD block, which encode MYB-related transcription factor, auxin-responsive protein, F-box protein, and CYP450, respectively. The relative expression of candidate genes in V030 and V071 vegetable soybean (for the plant height, pod number, and fresh pod weight of V030 were lower than those of the V071 strains) was significantly different, and these genes could be involved in plant growth and development via various pathways. Altogether, we identified four candidate genes for pod yield and plant height from vegetable soybean germplasm. This study provides insights into the genomic basis for improving soybean and crucial genomic resources that can facilitate genome-assisted high-yielding vegetable soybean breeding.

## 1. Introduction

Soybean (*Glycine max* (L.) Merr.) is a globally cultivated oil-seed crop. It is also an important source of high-quality vegetable protein [1]. Based on the different harvesting times, soybeans can be divided into two crop types: grain soybean and vegetable soybean (edamame). Vegetable soybeans are harvested at the R6 stage, when the pods are green and the seeds are fully developed [2]. The large pods and large grains are two important visual qualities of vegetable soybeans [3,4]. Therefore, pod quality has long been considered one of the most important traits in vegetable soybean breeding. Additionally, vegetable soybean yield is known to be affected by plant height, pod number, pod width, pod thickness, pod length, and fresh pod weight per plant. With the accelerated development of the global economy and population growth, being an inexpensive source of vegetable protein and oil, the demand for vegetable soybeans has sharply increased [5,6,7], but fewer reports focus on the yield of vegetable soybean compared to grain soybean at present. Therefore, it is necessary to analyze the genetic basis of soybean yield in R6 (the mature stage of the vegetable soybean) and to improve the overall yield of vegetable soybean crops.

Understanding the genetic variations related to yield traits is necessary for successful plant breeding [8]. Planting architecture is a key factor affecting soybeans’ planting density and seed yield. The ideal soybean plant structure not only optimizes the structure of the canopy but also prevents lodging, improves photosynthetic efficiency, and achieves a higher yield [7,8]. In terms of crop improvement, vegetable soybeans are assessed based on soybean yield per plant, pod number, pod length, pod width, pod thickness, plant height, and branch number. Therefore, identifying genetic loci associated with yield-related traits could contribute to the breeding of high yielding vegetable soybean. Furthermore, it could improve our understanding of the complex interaction of yield-related traits and working molecular mechanisms to improve vegetable soybean yield [9,10,11,12].

Genome-wide association studies (GWAS) are a highly effective method for dissecting the natural variation of quantitative traits based on linkage disequilibrium, which can provide a theoretical basis for analyzing the genetic structure and molecular pathways for the improvement of complex soybean traits [11,13,14]. In one such study, 125 candidate selection regions and 5 potential candidate genes related to agronomic traits were predicted using GWAS [15]. Zhang et al. identified 22 loci with minor effects and predicted 3 important candidate genes on chromosome 19 using the results of GWAS [16]. In another study, a total of 58 SNP_S_ significantly correlated with seed weight, internode number, and plant height, were identified using GWAS, and 28 candidate genes related to yield traits were predicted [17]. In addition, 27 quantitative trait nucleotides (QTNs) associated with seed size correlations were identified using GWAS [18,19]. Although the plant architecture and yield traits of soybean have been identified, mining yield trait-related genes in vegetable soybean (the mature stage of vegetable soybeans) has rarely been reported. Thus, quantitative genetics can identify genomic regions associated with yield traits, which could facilitate the breeding of improved and higher-yielding soybean cultivars.

Considering the gaps in the current research, we collected and evaluated 188 diverse vegetable soybean genotypes for 6 yield-related traits: the number of pods per plant, plant height, fresh pod weight, pod width, pod thickness, and pod length. Furthermore, GWAS was used to identify genetic loci and candidate genes for yield traits. The genetic network underlying the six traits through the LD of the linked loci with significant phenotypic correlation was further characterized. These results provide an important theoretical understanding of the genetic basis of yield traits and will facilitate the future breeding of vegetable soybean.

## 2. Results

### 2.1. Phenotype Variations of Yield-Related Traits in Vegetable Soybean

**Figure 1:**
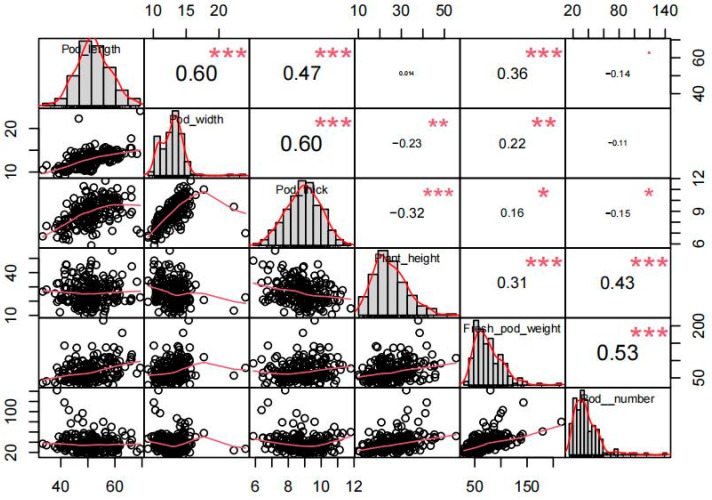
Correlation analysis of 188 vegetable soybean accessions and yield traits. One-way ANOVA was used to generate the *p* values. (*, **, *** indicate *p* < 0.05, 0.01, 0.001, respectively).

To identify important yield-related candidate genes from vegetable soybean germplasm resources, we conducted whole-genome re-sequencing and GWAS on a panel of 188 vegetable soybean accessions. Firstly, six yield-related traits of vegetable soybeans were investigated in the R6 stage: plant height, fresh pod weight per plant, pod length, number of pods per plant, pod width, and pod thickness. Among them, the vegetable soybean accessions V071 (plant height: 55.45 cm; fresh pod weight per plant: 215.67 g; number of pods per plant: 80) had higher yield-related traits than V030 (plant height: 10.10 cm; fresh pod weight per plant: 29.3 g; number of pods per plant: 5). Meanwhile, it can be seen that the variation coefficients of pod length (13.22%), pod width (14.65%), and pod thickness (12.75%) were relatively small, whereas the variation coefficients of the number of pods per plant (19.41%), plant height (32.08%), and fresh pod weight per plant were relatively moderate (40.91%) (Table 1). The average values of yield-related traits for vegetable soybeans during the R6 period analyzed conform to the normal distribution of data (Figure 1). 

### 2.2. Population Structure Analysis of Vegetable Soybean

**Figure 2:**
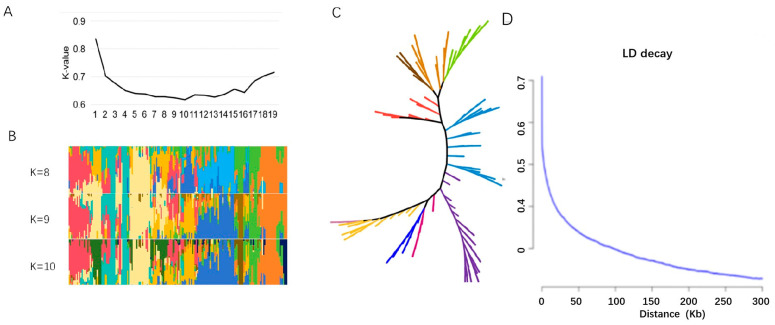
Population structure and linkage disequilibrium analysis of 188 vegetable soybeans. (**A**). Cross validation error rate for 188 samples based on clustering. (**B**). Bar plot divides the population of 188 vegetable soybeans into 10 cluster and every color represents a cluster. (**C**). Evolutionary tree of 188 soybean germplasm. (**D**). LD analysis for the 188 vegetable soybean germplasm.

Further genetic differentiation analysis was conducted for soybean accessions obtained from North and South China, and as well as from other countries. An adjacent tree shows that genotypes are divided into about nine different groups (Figure 2A–C). In addition, 66,825 high quality SNPs were used to evaluate population structure across the panel with K values ranging. LnP (D) values continued to increase and significant changes were observed as the K-value changed from 9 to 10 (Figure 2A,B). Thus, the most likely value of K is 9, and this division of the soybean panel is consistent with a significant delta K value (Figure 2A,B). Furthermore, this is consistent with the adjacent phylogenetic tree (Figure 2C). In addition, 188 vegetable soybean accessions were assessed for linkage disequilibrium (LD) using a subset of high-quality markers. At a threshold of R^2^ = 0.317, the average decay distance of LD was 150.00 kb for all 188 vegetable soybean accessions (Figure 2D).

### 2.3. Yield-Related SNPs Were Identified in Vegetable Soybean via GWAS

**Figure 3:**
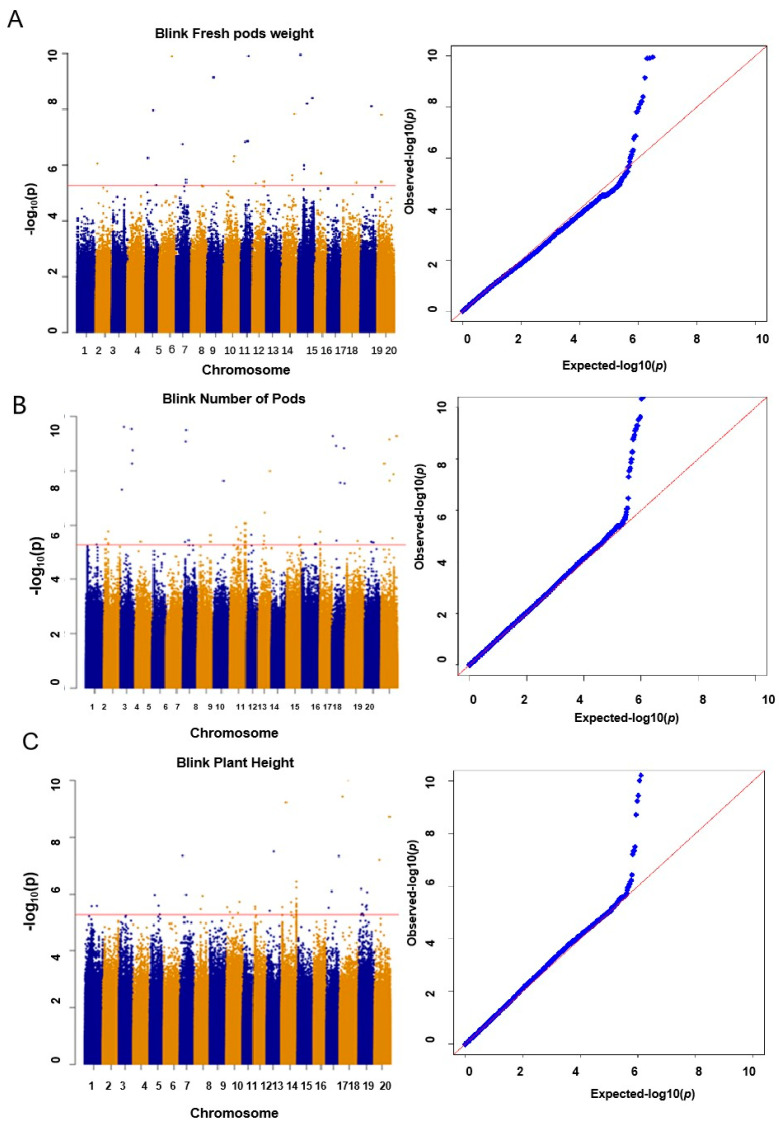
Genome-wide association analysis Manhattan plots and QQ plots of 188 vegetable soybean accession for the fresh pod weight (**A**), number of pods (**B**), and plant height (**C**).

The BLINK model was used to analyze the yield traits of 188 vegetable soybean accessions. A total of 116 significant yield-related SNPs were identified, and the *p*-values were less than 0.00001 (Figure 3 and Table 2). For fresh pod weight, 45 SNPs were detected and located on chromosomes 2, 5, 6, 7, 9, 10, 11, 14, 15, 16, 18, 19, and 20 (Figure 3A and Table 2); among them, three significantly related SNPs spanning a region of approximately 1.35 Mb (36.20 Mb to 37.55 Mb) were juxtaposed in the Chr10:37341548-37690972 [20]. In addition, a total of 8 SNPs associated with fresh pod weight on the 6, 9, 11, and 15 chromosomes were identified. Among them, one significant SNP (ss18491673) was 0.78 Mb away from the known SNP (ss17708693) on chromosome 9. Interestingly, the ss17708693 has been reported to regulate the soybean seed weight [21]. Meanwhile, 35 SNPs associated with pod number were identified and located on chromosomes 3, 7, 10, 12, 17, and 20 (Figure 3B and Table 2), and a total of 6 significantly related SNPs related to soybean yield spanning a region of approximately 8.45 Mb (31.00 Mb to 39.50 Mb) were juxtaposed on Chr03:3343094 [22]. For plant height, 36 SNPs were identified and located on chromosomes 1, 5, 7, 8, 10, 12, 13, 14, 17, 19, and 20 (Figure 3C and Table 2). Among them, one SNP was located 0.96 Mb away from the AP2 domain encoding gene (Glyma.07g078600) on chromosome 7, which is a large class of plant specific transcription factor that plays an important role in many biological processes, such as growth, development, and abiotic stress responses [23,24]. In addition, a total of seven significantly related SNPs spanning a region of approximately 2.5 Mb (21.00 Mb to 23.5 Mb on chromosome 13) were juxtaposed with the SWI/SNF locus (Glyma13g118200), which relates to growth and development [24,25].

### 2.4. Candidate Gene Analysis of Yield-Related Traits in Vegetable Soybean

According to the results of GWAS, ss39469452, ss8350061, ss18491673, ss36769025, and ss22150035 were located in the vicinity of known QTL intervals [20,21,22,23,24,25]. For 5 SNPs, genes located in 200 kbp genomic regions of each significant SNP were identified as candidate genes. A total of 56 candidate genes were identified under these significant SNPs. Phytozome 13 and Dicots Plaza 5.0 was used to analyze the function of these genes. Of these genes, 11 were found to be functionally annotated genes, while the remaining genes were hypothetical proteins with no functional annotation. Among these functionally annotated genes, Glyma.03G183200, an auxin-responsive family protein located on chromosome 3 near the significant SNP (ss39469452) for pod number. Candidate genes located near peak SNPs for fresh pod weight were *Glyma.09G102300* and *Glyma.09G102200*, encoding F-box protein and CYP family protein, respectively. Glyma.13G109100 was located near the significant SNP (ss22150035) on the chromosome, which encodes MYB-related transcription factors.

**Figure 4:**
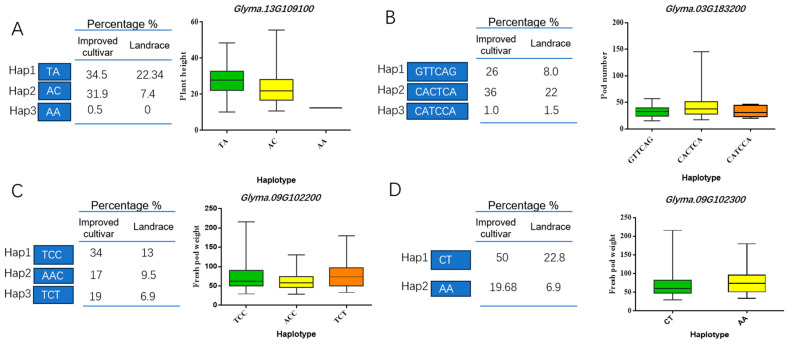
Haplotype analysis for the yield-related traits and candidate genes in 188 vegetable soybeans. (**A**). Haplotype analysis for the plant height and candidate genes of vegetable soybean (*p* < 0.05); (**B**). Haplotype analysis for the pod number and candidate genes of vegetable soybean (*p* < 0.05); (**C**,**D**). Haplotype analysis for the fresh pod weight and candidate genes of vegetable soybean (*p* < 0.05).

**Figure 5:**
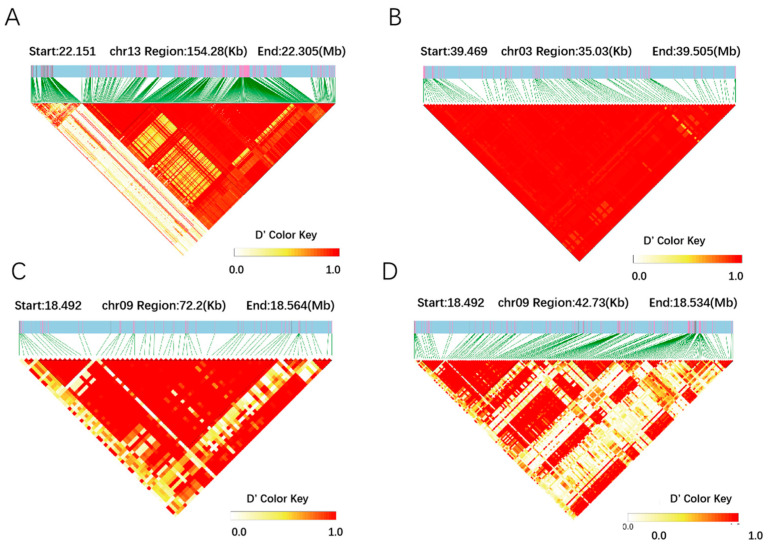
Linkage disequilibrium analysis for the candidate genes related to vegetable soybean yield traits. (**A**). Linkage disequilibrium analysis for the plant height-related candidate genes (*Glyma.13g109100)* in the 188 vegetable soybeans. (**B**). Linkage disequilibrium analysis for the pod number-related candidate genes (*Glyma.03g183200*) in the 188 vegetable soybeans. (**C**, **D**). Linkage disequilibrium analysis for the fresh pod number-related candidate genes (*Glyma.09g102200* and *Glyma.09g102300*) in the 188 vegetable soybeans.

To further explore the key candidate genes for vegetable soybean yield traits, a haplotype analysis of the above 11 candidate genes was performed (Figure 4). Importantly, the plant height-related candidate gene *Glyma.13g109100* identified has three haplotypes. Further analysis depicted that 22.34% of landraces and 34.5% of improved cultivars possessed Hap1(TA), which had a higher plant height than Hap3(AA). The number of pods per plant-related candidate gene *Glyma.03g183200* also had three haplotypes; interestingly, 8% of landraces and 26% of improved cultivars possessed Hap1(GTTCAG), which produced a higher number of pods per plant than Hap3(CATCCA). Simultaneously, two haplotypes were identified for the fresh pod weight-related candidate genes. Among these two genes, *Glyma.09g102200* was identified as having three haplotypes: 13% of landraces and 34% of improved cultivars possessed Hap1(TCC), which had a greater fresh pod weight than did cultivars with Hap3(TCT); *Glyma.09g102300* was identified as having two haplotypes: 22.8% of landraces and 50% of improved cultivars possessed Hap1(CT), which had a greater fresh pod weight per plant than did cultivars with Hap3(AA). Meanwhile, LD linkage disequilibrium analysis was performed on the above candidate genes, and it was found that the above genes were all in the LD block (Figure 5). It was further indicated that *Glyma.13g109100*, *Glyma.03g183200*, *Glyma.09g102200*, *and Glyma.09g102300* could be candidate genes to regulate the vegetable soybean plant height and fresh pod weight traits.

### 2.5. Different Expression Pattern of Candidate Genes in Pods and Stem

**Figure 6:**
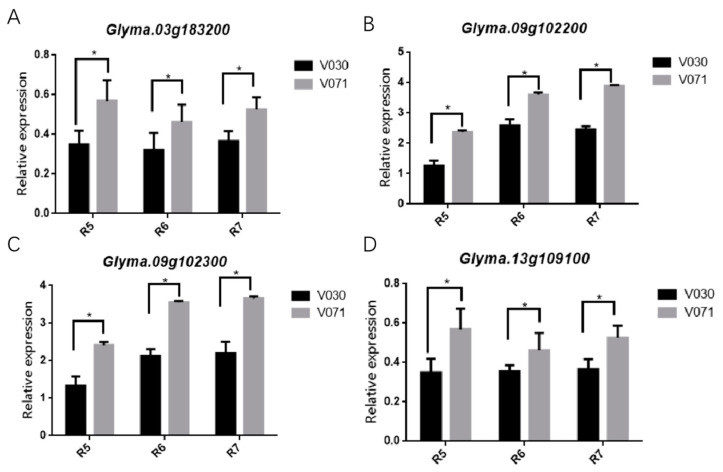
Expression analysis of potential candidate genes in V030 and V071 at three growth developmental stages. (**A**). Relative expression analysis of vegetable soybean pod number-related candidate gene of *Glyma.03g183200*. (**B**,**C**). Relative expression analysis for the vegetable soybean fresh pod weight-related candidate gene of *Glyma09g102200* and *Glyma09g102300*. (**D**). Relative expression analysis for the plant height-related candidate genes of *Glyma.13g109100* (* indicate *p* < 0.05).

In order to confirm whether candidate genes could regulate the vegetable soybeans height, fresh pod weight, and pod number, the expression patterns of candidate genes were tested via qRT-PCR between V30 and V71 (Figure 6). The relative expression of the candidate genes of *Glyma.03g138200* were significantly different between V071 and V030 at the R5, R6, and R7 stages (*p* <  0.05), indicating that *Glyma.03g138200* gene was involved in the positive regulation of seed number (Figure 6A). The relative expression of the potential candidate genes associated with fresh pod weight, *Glyma.09g102200* and *Glyma.09g102300*, showed significant expression differences between V071 and V030 at the R5, R6, and R7 stages (*p* < 0.05). With the increase in the growth period, the expression level of these two genes also increased (Figure 6B,C). Therefore, we speculate that *Glyma.09g102200* and *Glyma.09g102300* play a crucial role in the pod filling period of vegetable soybean seeds. In addition, the plant height-related candidate gene *Glyma.13g109100* showed significantly different expression levels between V071 and V030 at the R6 stage (*p* < 0.05), indicating that *Glyma.13g109100* gene may have been involved in growth and development and positively regulate plant height (Figure 6D). Therefore, we supposed that the candidate genes of *Glyma.13g109100*, *Glyma.03g183200*, *Glyma.09g102300*, and *Glyma.09g10220000* have the ability to regulate vegetable soybean plant height, pod number, and fresh pod weight. 

## 3. Discussion

In Asia, soybeans are a widely consumed type of legume vegetable, especially in Japan and China. Owing to the superior nutrition, appearance, and taste of vegetable soybean, there is growing demand, globally, for the cultivation of this type of soybean. Since the 1990s, the market for vegetable soybeans in the United States has increased, reaching 10,000 tons in 2000 [29,30]. However, the demand for vegetable soybeans cannot be met due to the lack of high-yielding soybean varieties. China, the country of origin for soybeans, has the most enormous soybean genetic resources worldwide. Thus, we analyzed the genetic structure of vegetable soybeans and GWAS was used to identify beneficial genes for molecular breeding. 

The GWAS method was used to analyze and calculate the association between genotype and phenotype variation, and to dissect the genetic basis of important traits [31]. For instance, in the study of maize, the phenotypic variation of grain yield per plants and tassel branch number was reported as 42.37% and 49.79%, respectively; the coefficient of variation with grain width and 100-kernel weight was 11% and 19%, respectively [32]. Additionally, plant structure, especially the number of nodes and branches, largely determines the pod number and yield of soybeans. Furthermore, other plant structures, including the number and size of pods, are also major components affecting the structure of the plant and grain yield. Therefore, it is of great significance to explore candidate genes for yield-related traits in vegetable soybeans to improve soybean yield through precise genetic engineering and breeding.

In this study, 188 vegetable soybean accessions were divided into 9 categories using population structure analysis, which indicated some variation within the population [33]. In addition, during population analysis, the acceptable distance between candidate genes and markers was determined by LD, and the LD varied among different populations [34]. The LD decay distance of the 188 vegetable soybean varieties in this study was 150.00 kb (r^2^ = 0.375), which is within the previously reported range (90–574 kb) [35]. In a previous study, the QTL region related to plant height was located on ss21,937,082-ss23,937,081 in the Chr13 [23,24], ss22150035 was located in this interval, and *Glyma.13G109100* was located on chromosome 13 near the significant SNP ss22150035 for plant height. In addition, *Glyma.13G109100* showed higher expression levels in the long plant height variety than in the short height variety (Figure 6), indicating that it may be involved in regulating the vegetable soybean plant height. This gene encodes MYB-related transcription factors, a highly conserved transcription factor family in eukaryotes, which is involved in many developmental processes such as root hair development, pollen formation, seed germination, flower stem strength, and yield. It also plays a role in abiotic stresses such as drought, ultraviolet light, cold stress, high temperature stress, and salt stress [36,37]. Meanwhile, MYB-related transcription factors could also reduce cell size and regulate plant structure, such as internode length, petiole length, leaf area, and plant height via the brassinosteroid (BR) pathway [36]. Other studies have also shown that *OsMYB110* not only changes plant height but also endows rice yield by increasing the number of grains per panicle and grain size [28]. Therefore, *Glyma.13G109100* was predicted to be a candidate gene that regulates vegetable soybean plant height.

In a recent report, 294 seed weight-related SNPs were identified in soybean chromosomes (http://www.soybase.org/) [16,38]. In addition, some identified SNP_S_ have been identified to correlate with yield traits, such as seed size, maturity, flowering time, and plant height [39]. For the seed weight, Copley et al. have shown that the SNP site of Chr09: 17708693, was important to regulate the seed weight, and was 0.78 Mb away from the SNP (ss18491673) on chromosome 9 in our study. Based on the annotation information, *Glyma.09G102300* and *Glyma.09G102200* were identified as a candidate gene to regulate vegetable soybean pod weight and seed yield. Among them, *Glyma.09G102300* mainly encodes F-box-related proteins, a family of proteins that exist widely in eukaryotes and contain the F-box domain, which plays an important role in the cell cycle, transcription, apoptosis, cell signal transduction, growth and development [40,41]. In addition, the F-box proteins, OsFBX206 and OsFBK12, could regulate ethylene synthesis and boisterousness to improve grain size and yield in rice and *Medicago truncatula* [42,43,44]. Meanwhile, we found that the NADH dehydrogenase1 alpha sub-complex was predicted to interact with *Glyma.09G102300*. The stability of NADH dehydrogenase1 has been demonstrated to play an important role in the regulation of seed germination, yield, and growth retardation in Arabidopsis and maize [45,46]. Moreover, the expression level of *Glyma.09G102300* in the V071 was higher than V030 in our study. Therefore, *Glyma.09G102300* can be regarded as a key candidate gene for regulating vegetable soybean pod weight.

Some subfamilies of CYP genes have been shown to influence both grain size and number [47,48]. In a recent report, *GmCYP78A72* was over-expressed in soybeans and proved that it could increase pod size, which is an extremely desirable trait for enhancing productivity [49]. In addition, other studies have shown that *TaCYP78A* participates in the auxin synthesis pathway and promotes auxin accumulation and cell wall remodeling through the transcriptome and hormone metabolome analyses [50]. These studies suggest that members of the CYP78A subfamily that encode cytochrome P450 generate growth signals that enhance plant fruit and seed (grain) development. Although the CYP subfamily is conserved in angiosperms, the contribution to the physiological function of the CYP72A subfamily is largely unknown. Recently, Gunupuru et al. identified that *TaCYP72A* had a positive effect on grain number in wheat [51]. In rice, the CYP724B1 could control grain size by affecting the expression level of BR-related genes [52,53]. Therefore, CYP72A also plays a positive regulatory role for grain weight. In this study, we identified a candidate gene related to the seed yield of vegetable soybean, *Glyma.09G102200*, which encodes CYP72A154 and belongs to the CYP72A subfamily. The expression level of *Glyma.09G102200* showed significant differences between the V030 and V071 vegetable soybean at the R5, R6, and R7 stages (the fresh pod weight of V030 was lower than that of the V071 accession). It is suggested that CYP72A154 may be a candidate gene to improve vegetable soybean seed weight. Therefore, *Glyma.09g102300* and *Glyma.09g102200* were predicted to be the potential candidate genes for regulating vegetable soybean pod weight. These results indicate that SNP–GWAS is a simple, effective, and robust technique for identifying candidate genes associated with complex yield-related traits in vegetable soybean.

## 4. Method

### 4.1. Plant Material and Field Experiments

The vegetable soybean in this study consisted of 188 different genotypes, including 115 and 58 genotypes originating from South and North China, and 14 genotypes from North America, India, Brazil, and Thailand. Of the 188 accessions, 44 were landraces and 144 were improved cultivars (Table S1). Field trials were conducted at Yazhou Bay Area (109.174313E, 18.352212N) in Hainan Province, China, in December of 2021 and 2022, respectively. A single-row plot randomized complete block design was used with three independent repeated planting experiments, respectively. As previously described, the soybean germplasm planting follows normal agronomic practices with some modifications [54]. A total of 60 vegetable soybean seeds were sown in three rows, 3 m in length, and spaced 0.5 m apart. Five randomly chosen plants from each row were recorded for phenotypic data at the R6 growth stage. Plant height was measured from above the surface of the soil to the tip of the main stem. Likewise, pod numbers were counted for every plant. The fresh pod weight was calculated with all the pods from every vegetable soybean. In addition, 10 pods were randomly selected from each plant to measure pod length, pod width, and pod thickness. 

### 4.2. Analysis of Vegetable Soybean Yield-Related Traits

The vegetable soybean yield traits measured in this study included plant height, pod number, fresh pod weight, per plant pod width, pod thickness, and pod length at the R6 stage. Correlation of vegetable soybean yield-related traits data were analyzed by R package (https://cran.r-project.org/web/packages/Hmisc/index.html, accessed on 5 March 2023). The standard deviation and coefficient of variation of vegetable soybean yield traits were calculated using SPSS16 software. Coefficient of variation = SD (standard deviation)/mean.

### 4.3. Genotypic Data

DNA was extracted from vegetable soybean leaves using CTAB (hexadecyl trimethyl ammonium bromide, C8440, Solarbio, Beijing, China) [30]. At the same time, DNA libraries were prepared by the restriction enzyme ApeKI (R0643S, NEB, Ipswich, MA, USA) and sequenced [55,56]. At the Shenzhen Huada Gene Research Institute, we obtained SLAF tags by digestion of each soybean accession leaf’s DNA, fragment ligation, PCR amplification, and selection of target fragments for SLAF libraries. The Illumina HiSeqTM 2500 platform (Illumina, Inc., San Diego, CA, USA) was performed to generate 45 bp sequence reads at both ends of the sequencing tags from each accession library. The alignment of the obtained raw paired end reads with the reference genome (*Glycine max* Wm82. a2. v1) was performed using BWA software (Version: 0.6.1-r104). SAMtools 48 software was employed to convert the mapping results into the BAM format and to effectively filter out unmapped and non-unique reads. The BEDtools software (Version: 2.17.0) was utilized to calculate the sequence alignment coverage. The detection of SNPs was performed using the GATK (version 2.4-7-g5e89f01) and SAMtools software. The SNP annotation was conducted and analyzed based on the soybean genome (*Glycine max* Wm82. a2. v1) using the ANNOVER package.

### 4.4. Population Analysis

The 188 accessions of soybean were re-sequenced and approximately 10 Gb of raw data were collected from each genome. TRIMMOMATIC (v.0.36) was used to filter the adaptors and low-quality bases of the raw data, and BOWTIE 2 (v.2.4.1) were used to map clean data to the Williams82.v2.1 genome with the default parameters [57]. Sentieon software (https://www.sentieon.com/) was used for the global realignment of reads and to produce VCF files. VCF tools were further employed to filter the raw VCF file with the following parameters (minDP 4, min-alleles 2, max-alleles 2, minQ 30, maxDP 60, max missing 0.9, maf 0.05), nucleotide diversity (p) (within a 500 kb window with no overlap), and Tajima’s D (with a 500 kb window with no overlap) [58]. Population structure analysis was performed using the admixture [59], and the best k (k = 9) was selected by STRUCTURE HARVESTER (v.2.3) [60]. A phylogenetic tree was constructed using the neighbor-joining method implemented in PHYLIP and displayed in iTOL [61,62]. PopLDdecay was used to analyze the population [63,64].

### 4.5. Genome-Wide Association Study and Candidate Gene Analysis

The phenotypic and genotypic data were used for GWAS. BLINK is an improved model version of fixed and random model cyclic probability unity (Farm CPU), which is statistically powerful and efficient in identifying significant SNPs associated with important traits [24]. Thus, the BLINK model was used to draw the Manhattan and QQ plot during the process of GWAS. Meanwhile, BLINK-generated data were used in R package [65]. All potential candidate genes were mined within 200 kb around the SNP sites. Gene expression and annotation analyses were performed using the Soybean Database (https://www.soybase.org/), which contains information on vegetable soybean yield-related traits.

### 4.6. Real-Time Quantitative Reverse Transcription PCR

To analyze the expression patterns of *Glyma.09G102300*, *Glyma.09G102200*, *Glyma.03g183200*, and *Glyma.13G109100* in V071 and V030, the pod and main stem at the R5, R6, and R7 periods were harvested, and RNA was extracted by using RNAiso plus (AJ31085A, TaKaRa, Japan). Total RNA (1 ug) was reverse transcribed into cDNA by Superscript IV reverse transcriptase (Thermo Fisher, Waltham, MA, USA). Real-time PCR amplification was performed in a 20 μL reaction: 1 μL cDNA, 0.2 mM of forward primer/reverse primer in 0.5 μL, 10 μL SYBR Green PCR Master Mix (Takara, Japan), and 8 μL ddH_2_O. The quantitative real-time PCR reaction conditions are as follows: 95 °C for 5 min, followed by 40 cycles at 95 °C for 10 s, and 62 °C for 25 s. The *GmEF1A* gene was used as an internal control, and the relative expression data were calculated using the method of 2^−∆∆CT^. The primers used are listed in Table S2.

## 5. Conclusions

In this study, we genotyped 188 soybean accessions and evaluated their yield-related traits. We identified 116 significant SNPs associated with 3 traits. Among them, five SNPs were close to the known QTL interval for the soybean plant height, pod number, and pod weight traits. Based on gene annotation and expression analyses, *Glyma.09G102300*, *Glyma.09G102200*, *Glyma.03g183200*, and *Glyma.13G109100* were identified as candidate genes related to vegetable soybean pod number, fresh pod weight, and plant height, respectively. The results suggest that yield-related traits are controlled by a complicated regulatory network and high-yield soybean genotypes may have different combinations of genetic networks. This study has provided a better understanding of the genetic architecture and could help to design high yielding vegetable soybean varieties through marker-assisted breeding, and by using forward and reverse genetic approaches.

## Figures and Tables

**Table 1:** Statistics and difference analysis of yield-related traits in the 188 vegetable soybean accessions.

Trait	Max	Min	Mean	SD	CV (%)
Pod length (um)	69.33	33.30	50.90	6.73	13.22%
Pod width (um)	24.04	9.19	12.97	1.90	14.65%
Pod thickness (um)	11.80	5.87	8.84	1.13	12.75%
Plant height (cm)	55.45	10.10	26.18	8.40	32.08%
Pods number	80.00	5.00	9.00	2.00	19.41%
Fresh pod weight (g)	215.67	29.30	70.34	28.78	40.91%

**Table 2:** List of significant SNPs associated with vegetable soybean yield-related traits and candidate genes.

Traits	Chr	SNP	−log10(P)	Candidate	Position (bp)	Gene Annotation	Known QTL/Ref.
Fresh pod weight	09	ss18491673	3 × 10^−5^	*Glyma.09G102300*	18,200,141–18,203,467	F-box protein	Seed weight/30-5 [21]
Fresh pod weight	09	ss18491673	3 × 10^−5^	*Glyma.09G101100*	18,049,793–18,052,768	WAT1-related protein	Seed weight/30-5 [21]
Fresh pod weight	09	ss18491673	3 × 10^−5^	*Glyma.09G101200*	18,059,406–18,066,433	Transcriptional regulator SNIP1	Seed weight/30-5 [21]
Fresh pod weight	09	ss18491673	3 × 10^−5^	*Glyma.09G101300*	18,077,704–18,089,366	Solute carrier family 35, member C2 (SLC35C2)	Seed weight/30-5 [21]
Fresh pod weight	09	ss18491673	3 × 10^−5^	*Glyma.09G102200*	18,535,131–18,535,371	CYP72A154	Seed weight/30-5 [21]
Fresh pod weight	10	ss36769025	5.73 × 10^−5^	*Glyma.10G135500*	36,601,462–36,604,173	F-box domain	[20]
Pod number	03	ss39469452	1.94 × 10^−15^	*Glyma.03G182100*	40,584,252–40,584,617	Small auxin-up RNA	[22]
Pod number	03	ss39469452	1.94 × 10^−15^	*Glyma.03G183200*	40,675,941–40,676,324	Auxin-responsive family protein	[22]
Plant height	13	ss22150035	3.17 × 10^−8^	*Glyma.13G107400*	21,210,684–21,224,769	Myosin-11-RELATED protein	[24,25]
Plant height	13	ss22150035	3.17 × 10^−8^	*Glyma.13G109100*	21,330,744–21,332,237	MYB-related transcription factors	[24,25,26]
Plant height	07	ss8350061	4.49 × 10^−8^	*Glyma.07G089000*	8,151,468–8,158,252	Vernalization-insensitive protein3	[23,27,28]

## Data Availability

Data are contained within the article.

## Supplementary Materials

The following supporting information can be downloaded at: https://www.mdpi.com/article/10.3390/plants13111442/s1, Table S1 Geographical source of 188 soybean germplasm in this study; Table S2 qRT-PCR Primer Squence list.

## References

[1] Liu N., Niu Y., Zhang G., Feng Z., Bo Y., Lian J., Wang B., Gong Y. (2022). Genome sequencing and population resequencing provide insights into the genetic basis of domestication and diversity of vegetable soybean. Hortic. Res..

[2] Kao C., He S., Wang C., Lai Z., Lin D., Chen S. (2021). A modified roger’s distance algorithm for mixed quantitative-qualitative phenotypes to establish a core collection for Taiwanese vegetable soybeans. Front. Plant Sci..

[3] Zhang B., Lord N., Kuhar T., Duncan S., Huang H., Ross J., Rideout S., Arancibia R., Reiter M., Li S. (2022). VT Sweet: A vegetable soybean cultivar for commercial edamame production in the mid-Atlantic USA. J. Plant Regist..

[4] Chen Z., Zhong W., Zhou Y., Ji P., Wan Y., Shi S., Yang Z., Gong Y., Mu F., Chen S. (2022). Integrative analysis of metabolome and transcriptome reveals the improvements of seed quality in vegetable soybean (*Glycine max* (L.) Merr.). Phytochemistry.

[5] Xu W., Liu H., Li S., Zhang W., Wang Q., Zhang H., Liu X., Cui X., Chen X., Tang W. (2022). GWAS and identification of candidate genes associated with seed soluble sugar content in vegetable soybean. Agron. J..

[6] Nair R.M., Boddepalli V.N., Yan M.R., Kumar V., Gill B., Pan R.S., Wang C., Hartman G.L., Souza S.E.R., Somta P. (2023). Global Status of Vegetable Soybean. Plants.

[7] Zhang H., Hao D., Sitoe H.M., Yin Z., Hu Z., Zhang G., Yu D. (2015). Genetic dissection of the relationship between plant architecture and yield component traits in soybean (*Glycine max*) by association analysis across multiple environments. Plant Breed.

[8] Zhao X., Dong H., Chang H., Zhao J., Teng W., Qiu L., Li W., Han Y. (2019). Genome wide association mapping and candidate gene analysis for hundred seed weight in soybean [*Glycine max* (L.) Merrill]. BMC Genom..

[9] Li X., Zhang X., Zhu L., Bu Y., Wang X., Zhang X., Zhou Y., Wang X., Guo N., Qiu L. (2019). Genome-wide association study of four yield-related traits at the R6 stage in soybean. BMC Genet..

[10] Cao Y., Jia S., Chen L., Zeng S., Zhao T., Karikari B. (2022). Identification of major genomic regions for soybean seed weight by genome-wide association study. Mol. Breed..

[11] Ayalew H., Schapaugh W., Vuong T., Nguyen H.T. (2022). Genome-wide association analysis identified consistent QTL for seed yield in a soybean diversity panel tested across multiple environments. Plant Genome.

[12] Wang J., Hu B., Jing Y., Hu X., Guo Y., Chen J., Liu Y., Hao J., Li W.X., Ning H. (2022). Detecting QTL and Candidate Genes for Plant Height in Soybean via Linkage Analysis and GWAS. Front. Plant Sci..

[13] Chang F., Guo C., Sun F., Zhang J., Wang Z., Kong J., He Q., Sharmin R.A., Zhao T. (2018). Genome-Wide Association Studies for Dynamic Plant Height and Number of Nodes on the Main Stem in Summer Sowing Soybeans. Front. Plant Sci..

[14] Liu Z., Li H., Fan X., Huang W., Yang J., Wen Z., Li Y., Guan R., Guo Y., Chang R. (2017). Phenotypic characterization and genetic dissection of nine agronomic traits in Tokachi nagaha and its derived cultivars in soybean (*Glycine max* (L.) Merr.). Plant Sci..

[15] Wen Z., Boyse J.F., Song Q., Cregan P.B., Wang D. (2015). Genomic consequences of selection and genome-wide association mapping in soybean. BMC Genom..

[16] Zhang J., Song Q., Cregan P.B., Jiang G.L. (2016). Genome-wide association study, genomic prediction and marker-assisted selection for seed weight in soybean (*Glycine max*). Theor. Appl. Genet..

[17] Assefa T., Otyama P.I., Brown A.V., Kalberer S.R., Kulkarni R.S., Cannon S.B. (2019). Genome-wide associations and epistatic interactions for internode number, plant height, seed weight and seed yield in soybean. BMC Genom..

[18] Zhao X., Li W., Zhao X., Wang J., Liu Z., Han Y., Li W. (2019). Genome-wide association mapping and candidate gene analysis for seed shape in soybean (*Glycine max*). Crop Pasture Sci..

[19] Zhang X., Ding W., Xue D., Li X., Zhou Y., Shen J., Feng J., Guo N., Qiu L., Xing H. (2021). Genome-wide association studies of plant architecture-related traits and 100-seed weight in soybean landraces. BMC Genom. Data.

[20] Niu M., Tian K., Chen Q., Yang C., Zhang M., Sun S., Wang X. (2024). A multi-trait GWAS-based genetic association network controlling soybean architecture and seed traits. Front. Plant Sci..

[21] Copley T.R., Duceppe M.O., O’Donoughue L.S. (2018). Identification of novel loci associated with maturity and yield traits in early maturity soybean plant introduction lines. BMC Genom..

[22] Wang Q., Zhang W., Xu W., Zhang H., Liu X., Chen X., Chen H. (2024). Genome-Wide Association Study and Identification of Candidate Genes Associated with Seed Number per Pod in Soybean. Int. J. Mol. Sci..

[23] Ma Z., Jin Y.M., Wu T., Hu L., Zhang Y., Jiang W., Du X. (2022). OsDREB2B, an AP2/ERF transcription factor, negatively regulates plant height by conferring GA metabolism in rice. Front. Plant Sci..

[24] Ravelombola W., Qin J., Shi A., Song Q., Yuan J., Wang F., Chen P., Yan L., Feng Y., Zhao T. (2021). Genome-wide association study and genomic selection for yield and related traits in soybean. PLoS ONE.

[25] Ojolo S.P., Cao S., Priyadarshani S.V.G.N., Li W., Yan M., Aslam M., Zhao H., Qin Y. (2018). Regulation of Plant Growth and Development: A Review from a Chromatin Remodeling Perspective. Front. Plant Sci..

[26] Yoon J., Jeong H.J., Baek G., Yang J., Peng X., Tun W., Kim S.T., An G., Cho L.H. (2021). A VIN3-like Protein OsVIL1 Is Involved in Grain Yield and Biomass in Rice. Plants.

[27] Yang J., Cho L.H., Yoon J., Yoon H., Wai A.H., Hong W.J., Han M., Sakakibara H., Liang W., Jung K.H. (2019). Chromatin interacting factor OsVIL2 increases biomass and rice grain yield. Plant Biotechnol. J..

[28] Wang T., Jin Y., Deng L., Li F., Wang Z., Zhu Y., Wu Y., Qu H., Zhang S., Liu Y. (2024). The transcription factor MYB110 regulates plant height, lodging resistance, and grain yield in rice. Plant Cell.

[29] Lin C. Frozen edamame: Global market conditions. Proceedings of the Second International Vegetable Soybean Conference.

[30] Zhang Q., Li. Y., Chin K.L., Qi Y. (2017). Vegetable soybean: Seed composition and production research. Ital. J. Agron..

[31] Yano K., Yamamoto E., Aya K., Takeuchi H., Lo P.C., Hu L., Yamasaki M., Yoshida S., Kitano H., Hirano K. (2016). Genome-wide association study using whole-genome sequencing rapidly identifies new genes influencing agronomic traits in rice. Nat. Genet..

[32] Zeng T., Meng Z., Yue R., Lu S., Li W., Li W., Meng H., Sun Q. (2022). Genome wide association analysis for yield related traits in maize. BMC Plant Biol..

[33] Jiao X., Lyu Y., Wu X., Li H., Cheng L., Zhang C., Yuan L., Jiang R., Jiang B., Rengel Z. (2016). Grain production versus resource and environmental costs: Towards increasing sustainability of nutrient use in China. J. Exp. Bot..

[34] Eltaher S., Sallam A., Belamkar V., Emara H.A., Nower A.A., Salem K.F.M., Poland J., Baenziger P.S. (2018). Genetic Diversity and Population Structure of F3:6Nebraska Winter Wheat Genotypes Using Genotyping-By-Sequencing. Front. Genet..

[35] Li Y.H., Reif J.C., Hong H.L., Li H.H., Liu Z.X., Ma Y.S., Li J., Tian Y., Li Y.F., Li W.B. (2018). Genome-wide association mapping of QTL underlying seed oil and protein contents of a diverse panel of soybean accessions. Plant Sci..

[36] Chen L., Yang H., Fang Y., Guo W., Chen H., Zhang X., Dai W., Chen S., Hao Q., Yuan S. (2021). Overexpression of GmMYB14 improves high-density yield and drought tolerance of soybean through regulating plant architecture mediated by the brassinosteroid pathway. Plant Biotechnol. J..

[37] Qi X., Tang W., Li W., He Z., Xu W., Fan Z., Zhou Y., Wang C., Xu Z., Chen J. (2021). Arabidopsis-Protein β Subunit AGB1 Negatively Regulates DNA Binding of MYB62, a Suppressor in the Gibberellin Pathway. Int. J. Mol. Sci..

[38] Li S., Cao Y., Wang C., Yan C., Sun X., Zhang L., Wang W., Song S. (2023). Genome-wide association mapping for yield-related traits in soybean (Glycine max) under well-watered and drought-stressed conditions. Front. Plant Sci..

[39] Sonah H., O’Donoughue L., Cober E., Rajcan I., Belzile F. (2015). Identification of loci governing eight agronomic traits using a GBS-GWAS approach and validation by QTL mapping in soya bean. Plant Biotechnol. J..

[40] Xu K., Zhao Y., Zhao Y., Feng C., Zhang Y., Wang F., Li X., Gao H., Liu W., Jing Y. (2022). Soybean F-Box-Like Protein GmFBL144 Interacts with Small Heat Shock Protein and Negatively Regulates Plant Drought Stress Tolerance. Front. Plant Sci..

[41] Bu Q., Lv T., Shen H., Luong P., Wang J., Wang Z., Huang Z., Xiao L., Engineer C., Kim T.H. (2014). Regulation of drought tolerance by the F-box protein MAX2 in Arabidopsis. Plant Physiol..

[42] Chen Y., Xu Y., Luo W., Li W., Chen N., Zhang D., Chong K. (2013). The F-box protein OsFBK12 targets OsSAMS1 for degradation and affects pleiotropic phenotypes, including leaf senescence, in rice. Plant Physiol..

[43] Zhou S., Yang T., Mao Y., Liu Y., Guo S., Wang R., Fang Y.G., He L., Zhao B., Bai Q. (2021). The F-box protein MIO1/SLB1 regulates organ size and leaf movement in Medicago truncatula. J. Exp. Bot..

[44] Sun X., Xie Y., Xu K., Li J. (2024). Regulatory networks of the F-box protein FBX206 and OVATE family proteins modulate brassinosteroid pathway to regulate grain size and yield in rice. J. Exp. Bot..

[45] Lee K., Han J.H., Park Y.I., Colas des Francs-Small C., Small I., Kang H. (2017). The mitochondrial pentatricopeptide repeat protein PPR19 is involved in the stabilization of NADH dehydrogenase 1 transcripts and is crucial for mitochondrial function and Arabidopsis thaliana development. New Physiol..

[46] Cai M., Li S., Sun F., Sun Q., Zhao H., Ren X., Zhao Y., Tan B.C., Zhang Z., Qiu F. (2017). Emp10 encodes a mitochondrial PPR protein that affects the cis-splicing of nad2 intron 1 and seed development in maize. Plant J..

[47] Wang X., Li Y., Zhang H., Sun G., Zhang W., Qiu L. (2015). Evolution and association analysis of GmCYP78A10 gene with seed size/weight and pod number in soybean. Mol. Biol. Rep..

[48] Zhou C., Lin Q., Ren Y., Lan J., Miao R., Feng M., Wang X., Liu X., Zhang S., Pan T. (2023). A CYP78As-small grain4-coat protein complex II pathway promotes grain size in rice. Plant Cell.

[49] Zhao B., Dai A., Wei H., Yang S., Wang B., Jiang N., Feng X. (2016). Arabidopsis KLU homologue GmCYP78A72 regulates seed size in soybean. Plant Mol. Biol..

[50] Guo L., Ma M., Wu L., Zhou M., Li M., Wu B., Li L., Liu X., Jing R., Chen W. (2022). Modified expression of TaCYP78A5 enhances grain weight with yield potential by accumulating auxin in wheat (*Triticum aestivum* L.). Plant Biotechnol. J..

[51] Gunupuru L.R., Arunachalam C., Malla K.B., Kahla A., Perochon A., Jia J., Thapa G., Doohan F.M. (2018). A wheat cytochrome P450 enhances both resistance to deoxynivalenol and grain yield. PLoS ONE.

[52] Zhou Y., Tao Y., Zhu J., Miao J., Liu J., Liu Y., Yi C., Yang Z., Gong Z., Liang G. (2017). GNS4, a novel allele of DWARF11, regulates grain number and grain size in a high-yield rice variety. Rice.

[53] Zhou Y., Xie Y., Cai J., Liu C., Zhu H., Jiang R., Zhong Y., Zhang G., Tan B., Liu G. (2017). Substitution mapping of QTLs controlling seed dormancy using single segment substitution lines derived from multiple cultivated rice donors in seven cropping seasons. Theor. Appl. Genet..

[54] Elshire R.J., Glaubitz J.C., Sun Q., Poland J.A., Kawamoto K., Buckler E.S., Mitchell S.E. (2011). A robust, simple genotyping-by-sequencing (GBS) approach for high diversity species. PLoS ONE.

[55] Sonah H., Bastien M., Iquira E., Tardivel A., Légaré G., Boyle B., Normandeau É., Laroche J., Larose S., Jean M. (2013). An improved genotyping by sequencing (GBS) approach offering increased versatility and efficiency of SNP discovery and genotyping. PLoS ONE.

[56] Bolger A.M., Lohse M., Usadel B. (2014). Trimmomatic: A flexible trimmer for Illumina sequence data. Bioinformatics.

[57] Langmead B., Salzberg S.L. (2012). Fast gapped-read alignment with Bowtie 2. Nat. Methods.

[58] Danecek P., Auton A., Abecasis G., Albers C.A., Banks E., DePristo M.A., Handsaker R.E., Lunter G., Marth G.T., Sherry S.T. (2011). The variant call format and VCFtools. Bioinformatics.

[59] Pritchard J.K., Stephens M., Donnelly P. (2000). Inference of population structure using multilocus genotype data. Genetics.

[60] Earl D.A., vonHoldt B.M. (2012). Structure Harvester: A website and program for visualizing STRUCTURE output and implementing the Evanno method. Conserv. Genet. Resour..

[61] Felsenstein J. (2005). PHYLIP (Phylogeny Inference Package) Version 3.6.

[62] Letunic I., Bork P. (2021). Interactive Tree of Life (iTOL) v5: An online tool for phylogenetic tree display and annotation. Nucleic Acids Res..

[63] Sabeti P.C., Reich D.E., Higgins J.M., Levine H.Z., Richter D.J., Schaffner S.F., Gabriel S.B., Platko J.V., Ptterson N.J., Lander E.S. (2002). Detecting recent positive selection in the human genome from haplotype structure. Nature.

[64] Zhang C., Dong S.S., Xu J.Y., He W.M., Yang T.L. (2019). PopLDdecay: A fast and effective tool for linkage disequilibrium decay analysis based on variant call format files. Bioinformatics.

[65] Huang M., Liu X., Zhou Y., Summers R.M., Zhang Z. (2019). BLINK: A package for the next level of genome-wide association studies with both individuals and markers in the millions. Gigascience.

