# Supplementary material for: Genome-Wide Association Analysis of Yield-Related Traits and Candidate Genes in Vegetable Soybean
Source: Plants (Basel). 2024 May 23;13(11):1442. doi: 10.3390/plants13111442 (PMC11174663; doi:10.3390/plants13111442)
Supplement: Supplementary file 1 [file plants-13-01442-s001.zip › plants-2969467-supplementary.pdf]

Table S1 Geographical source of 188 soybean germplasm in this study

Geographical source		Landrace	Improved cultivar	Total
South,China	Zhejiang	1	23	24
	Jiangsu	3	18	21
	Hainan	0	6	6
	Hubei	0	2	2
	Fujian	0	8	8
	Nanjing	0	7	7
	Shanghai	2	3	5
	Gansu	1	0	1
	Hunan	1	0	1
	Taiwan	0	4	4
	Yunnan	6	0	6
	Miandian	2	0	2
	Sichuan	1	5	6
	Guangdong	3	7	10
	Guangxi	10	2	12
North,China	Jilin	0	2	2
	Liaoning	7	32	39
	Heilongjiang	0	4	4
	Beijing	0	3	3
	Shandong	1	1	2
	Henan	1	0	1
	Shanxi	3	1	4
	Hebei	1	0	1
	Anhui	0	2	2
Other country	Nepal	1	0	1
	India	3	0	3
	American	3	0	3
	Switzerland	1	0	1

Brazil	5	0	5
Thailand	1	0	1
Serbia	1	0	1
Total	58	130	188

Table S2 qRT-PCR Primer Squence list

Gene	Primer sequence
Glyma.03G183200-F	CTCTATTGAGTTGGAGTTTTTG
Glyma.03G183200-R	AATTCTCTTGAGGTCATCTGGT
Glyma.13G109100-F	CCTCAATCTCCCTATCTCCTCC
Glyma.13G109100-R	CTAATTACACCATCCACAAGCC
Glyma.09g102300-F	TCATCATTTCCTTGCTTCCATT
Glyma.09g102300-R	AAACTTTTCCACCTCACCTACT
Glyma.09g102200-F	GAAGTAGTTATGAAGAAGGAAG
Glyma.09g102200-R	TTTCAATAAAGTTTGTAATGTG
